# Spontaneous bilateral subclavian vein thrombosis in a 40-year-old man: A case report

**DOI:** 10.1097/MD.0000000000010327

**Published:** 2018-04-13

**Authors:** Chun-Yen Huang, Yen-Hung Wu, I-Jeng Yeh, Yun-Yi Chen, Fung-Ya Kung

**Affiliations:** a Department of Emergency Medicine, Kaohsiung Medical University Hospital; b School of Medicine, College of Medicine, Kaohsiung Medical University, Kaohsiung, Taiwan.

**Keywords:** bilateral subclavian vein thrombosis, Paget–Schroetter syndrome

## Abstract

**Rationale:**

Paget–Schroetter syndrome (PSS) is an uncommon condition that refers to primary (spontaneous) thrombosis of the deep veins that drain the upper extremities because of anatomical anomalies or repetitive strenuous arm activity. Bilateral spontaneous upper extremity deep-vein thrombosis (UEDVT) is an extremely rare phenomenon in adults, which may be misdiagnosed by physicians in acute settings.

**Patient concerns:**

A 40-year-old man presented to our emergency department because of progressive left upper arm swelling for 1 day. He denied fever, chest pain, dyspnea, trauma, or any other systemic disease before. The swollen left arm also had no local heat or redness with normal radius pulsation. He was a laborer who lifted heavy objects.

**Diagnoses:**

Blood examination included tests for complete blood count, renal function, liver function, blood coagulation profile, cardiac enzyme levels, and D-dimer level. Results showed that the white blood cell count, renal and liver functions, and cardiac enzyme levels were all within their normal ranges, except for the elevated D-dimer level (1.93 mg/L). Chest radiography and electrocardiography were performed with nonspecific findings. Subsequently, computed tomographic angiography was recommended for the suspected deep-vein thrombosis. The report showed venous thrombosis involving the bilateral subclavian and internal jugular veins.

**Interventions:**

Heparin and enoxaparin were prescribed for this patient, with loading and maintenance doses. He was then admitted to our cardiovascular ward for further treatment.

**Outcomes:**

The patient was discharged 9 days later in a stable condition.

**Lessons:**

Emergency physicians should consider the rare condition of UEDVT when a healthy patient presents with acute arm swelling. Patient history taking should be thorough, especially concerning the risk factors of secondary causes and possible frequent vigorous heavy lifting and overhead motion. Without secondary risk factors, primary upper deep-vein thrombosis might be suspected. Further laboratory tests and imaging studies, especially bilateral imaging, should be arranged to exclude secondary causes and to confirm the diagnosis.

## 1 Introduction

Paget–Schroetter syndrome (PSS) is an uncommon condition that refers to primary (spontaneous) thrombosis of the deep veins that drain the upper extremities because of anatomic anomalies or repetitive strenuous arm activity.^[1]^ Bilateral spontaneous upper extremity deep-vein thrombosis (UEDVT) is an extremely rare phenomenon in adults, which may be misdiagnosed by physicians in acute settings. Here, we report a healthy 40-year-old man who was working as a blacksmith and presented with acute arm swelling diagnosed as spontaneous bilateral subclavian vein thrombosis. After we contacted the regulatory institutional review board of the Kaohsiung Medical University Hospital, ethical approval was not required for this case report article. Informed consent was obtained from the patient for the publication of this case report.

## 2 Case presentation

**Figure 1:**
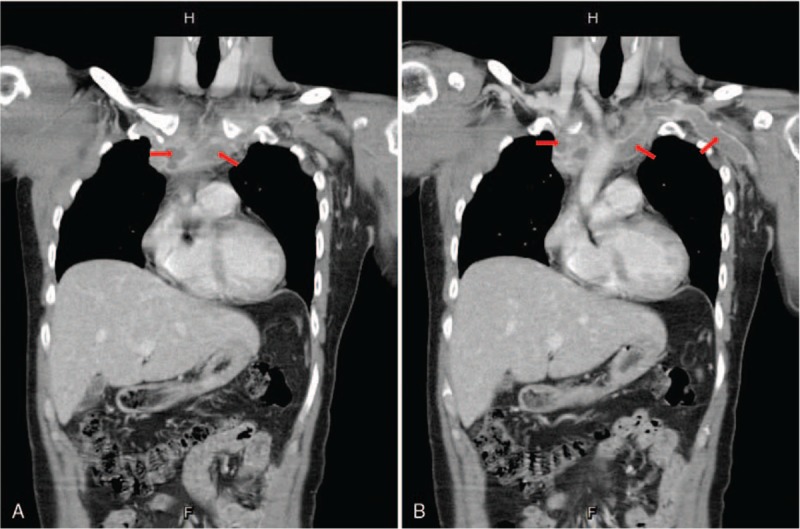
(A, B) Computed tomographic angiography image showing venous thrombosis involving bilateral subclavian veins.

A 40-year-old man presented to our emergency department because of progressive left upper arm swelling for 1 day. The status on arrival showed a heart rate of 104 beats per minute, blood pressure of 116/87 mm Hg, and body temperature of 36.5 °C, with clear consciousness. He denied fever, chest pain, dyspnea, trauma, or any other systemic disease before. The swelling in the left arm had no local heat or redness and had normal radius pulsation. He was a laborer who lifted heavy objects. Blood examination included complete blood count, renal function, liver function, blood coagulation profile, cardiac enzyme levels, and D-dimer level. Results showed that the white blood cell count, renal and liver functions, and cardiac enzymes were all within their normal ranges, except for the elevated D-dimer level (1.93 mg/L). Chest radiography and electrocardiography were performed with nonspecific findings. Then, computed tomographic angiography was recommended for the suspected DVT. The report showed that the venous thrombosis involved the bilateral subclavian and internal jugular veins (Fig. 1A and B). Heparin was prescribed for this patient at a loading dose (5000-unit intravenous injection) and maintenance dose (shift to enoxaparin 60-mg injection twice per day). He was then admitted to our cardiovascular ward for further treatment. After admission, we maintained the anticoagulation therapy (enoxaparin 60-mg twice per day for 4 days and shifted to 15-mg oral rivaroxaban twice per day until discharge). We surveyed possible causes but found no evidence of hypercoagulation disorder or antiphospholipid syndrome. During the admission period, hepatitis was found 2 days after admission (aspartate aminotransferase: 338 IU/L and alanine aminotransferase: 330 IU/L), without evidence of hepatitis B or C virus flare-up. Abdominal echography revealed no specific findings. The hepatitis condition improved after administration of silymarin (150 mg 3 times per day), and the patient was discharged 9 days later in a stable condition. The patient was followed up at the outpatient department (duration: initial 1 time per week and follow-up: 1 time per month) with medication of rivaroxaban 15 mg twice per day and plan to use the drug for at least 6 months. The patient did not have limb swelling or other venous thrombosis event after that.

## 3 Discussion

DVT and pulmonary embolism are 2 clinical conditions of venous thromboembolism (VTE). In all DVT patients, UEDVT accounts for only 4% to 10%.^[2,3]^ The etiology of UEDVT can be further divided into primary and secondary. Most UEDVT cases are secondary, which includes placement of a central venous catheter, malignancy, thrombophilia, surgery, or trauma of the shoulder and rheumatologic disorder.^[4,5]^ The annual incidence of primary (spontaneous) UEDVT is estimated at only 1 to 2 cases per 100,000 population,^[6]^ and bilateral primary UEDVT as in our patient is even rarer. Only few cases of bilateral UEDVT have been reported, and most of them are due to secondary causes such as ICD implantation,^[7]^ systemic sclerosis,^[8]^ Bechet disease,^[9]^ factor V Leiden heterozygote,^[10]^ and acquired immunodeficiency syndrome.^[11]^

PSS, defined as UEDVT due to anatomic anomalies compressing the axillosubclavian veins from the thoracic outlet, is the most common cause of primary UEDVT. Currently, it is thought to be due to not only the structural compression from the thoracic outlet but also repetitive strenuous arm movement, which leads to microtrauma of the veins and subsequent inflammation, fibrosis, and thrombus formation.^[5,12]^ PSS most commonly affects athletic males in their early thirties, with clinical manifestations of acute onset of upper extremity pain and swelling.^[13,14]^ Diagnosis is made on the basis of the above-mentioned typical history and by ultrasonography initially. Catheter-based venography is the standard diagnostic criterion, but computed tomographic venography is a safe alternative. Our patient was a healthy 40-year-old man who worked as a blacksmith. He needed to perform repetitive heavy lifting and overhead motion during work. He presented to our emergency department because of acute left arm swelling and pain, but computed tomographic venography revealed bilateral subclavian vein thrombosis. During hospitalization, further workup was performed and excluded possible malignancy, rheumatologic disorders, prothrombotic states, and acquired immunodeficiency syndrome. Finally, a diagnosis of bilateral spontaneous subclavian vein thrombosis was made.

The results of the laboratory tests for primary UEDVT were generally normal, except for the elevated D-dimer level, which is useful for excluding thrombosis as an etiology.^[15]^ Our patient had an elevated D-dimer level. In addition, his alanine transaminase (ALT) and aspartate transaminase (AST) levels were elevated, but not his creatinine kinase level. The result could not be explained by vigorous activity-induced rhabdomyolysis. No data on previous AST or ALT levels were available for comparison, and his test results for hepatitis virus infection and abdominal sonography were normal. His AST and ALT levels progressively declined as his symptoms improved, which implies some relationship between primary UEDVT and elevated AST and ALT levels. We found only one case report that discussed elevated transaminase level with primary UEDVT.^[16]^ Further study is needed.

Currently, anticoagulation is recommended for all patients diagnosed as having UEDVT^[17]^; however, no consensus has been reached for managing bilateral primary UEDVT. Aggressive treatment with thrombolysis, thoracic outlet decompression, or both is mainly based on symptom severity, duration, anatomical abnormalities, and surgical risks.^[12]^ Our patient received only anticoagulation therapy, and his symptoms gradually improved. Owing to the improvement of his clinical condition, further aggressive treatments were not required. We shifted intravenous anticoagulation to oral rivaroxaban as outpatient treatment for at least 3 months^[17]^ and continued follow-up in the cardiovascular outpatient department to prevent the recurrent thrombosis and postthrombotic syndrome.

## 4 Conclusion

Emergency physicians should consider the rare condition of UEDVT when a healthy patient presents with acute arm swelling. Patient history taking should be thorough, especially concerning the risk factors of secondary causes and possible frequent vigorous heavy lifting and overhead motion. Without secondary risk factors, primary upper DVT might be suspected. Further laboratory tests and imaging studies, especially bilateral imaging, should be arranged to exclude secondary causes and to confirm the diagnosis. Patients with this condition should receive anticoagulation therapy and require hospital admission for specialist evaluation as to the necessity for aggressive treatment.

## Author contributions

**Investigation:** Fung-Ya Kung.

**Resources:** Yun-Yi Chen.

**Visualization:** I-Jeng Yeh, Yun-Yi Chen.

**Writing – original draft:** Chun-Yen Huang, Yen-Hung Wu.

**Writing – review & editing:** Chun-Yen Huang, Yen-Hung Wu.
